# Probiotic Beverage From Pineapple Juice Fermented With *Lactobacillus* and *Bifidobacterium* Strains

**DOI:** 10.3389/fnut.2019.00054

**Published:** 2019-05-09

**Authors:** Bao Toan Nguyen, Erika Bujna, Noemi Fekete, Anh T. M. Tran, Judit M. Rezessy-Szabo, Ram Prasad, Quang D. Nguyen

**Affiliations:** ^1^Faculty of Food Science, Research Centre of Bioengineering and Process Engineering, Szent István University, Budapest, Hungary; ^2^Amity Institute of Microbial Technology, Amity University Uttar Pradesh, Noida, India; ^3^School of Environmental Science and Engineering, Sun Yat-Sen University, Guangzhou, China

**Keywords:** pineapple, bifidobacteria, lactic acid bacteria, fermented juice, probiotics, prebiotics

## Abstract

Pineapple is an economically significant plant and the third most important fruit crop in the tropical and subtropical regions of the world. In this study, fermentation of pineapple juice with probiotic bacteria *Lactobacillus* and *Bifidobacterium* strains as well as changes of some properties in the beverage during storage were investigated. All tested strains exhibited good growth properties on pineapple juice without supplementation of any nutrient compounds. After 24 h fermentation, the cell counts of lactobacilli passed the level of 5^*^10^9^ cfu/ml, while the cell number of bifidobacteria reached a level of 10^9^ cfu/ml. The highest volumetric productivity (3.5^*^10^8^ cfu/ml^*^h) was observed in *L. plantarum* 299V. The ratios of lactic acids to acetic acids in the cases of *L. plantarum* 299V and *L. acidophilus* La5 were 5.37 and 9.91, respectively. In the case of *B. lactis* Bb-12, the concentrations of lactic acid and acetic acid were 6 mM and 23 mM in natural juices, and 15 and 21 mM in the case of supplementation with prebiotics at the 16^th^ h of fermentation, respectively. Additionally, supplementation with prebiotics at the initiation of fermentation resulted 7 mM lactic acid and 23 mM acetic acid at the end of fermentation. Fructose was the most preferred sugar for both lactobacilli and bifidobacteria. Both total phenolic content and antioxidant capacity increased slightly during fermentation and dropped during the storage period. The microbial population did not change significantly during the first month of storage. After the storage period (2 months), the probiotic bacteria lost about 0.11 log cfu/ml viability after treatment with 0.3% pepsin for 135 min, and a further 0.1 log cfu/ml after treatment with 0.6% bile salts. These values were 10 times higher than data from the fresh fermented pineapple juice. Our results are very promising and may serve as a good base for developing probiotic pineapple juice.

## Introduction

Today, the concept using foods to promote a state of well-being, improving health, and reducing the risk of diseases has become the new frontier in the nutrition sciences and related fields (1). Furthermore, this concept is particularly important in light of the increasing cost of health care, the steady increase in life expectancy, and the desire of elderly people for improved life quality. Moreover, the emphasis has moved from medication to prevention. In this context, the development and contribution of functional foods—prebiotics, probiotics and synbiotics—must receive attention and should be key pillars of the health care system. Functional foods not only act as traditional nutrients, but they also have some additional beneficial effects such as improving health status, preventing and/or reducing nutrition-related diseases, and promoting a state of physical and mental well-being (2). A wide range of foods have been fermented or enriched in probiotics to be evaluated as possible carriers of these beneficial microorganisms and successfully placed on the market. Several species of *Lactobacillus* and *Bifidobacterium* have become the most commonly used probiotic strains in these food products, but others such as *Saccharomyces cerevisiae* (*boulardii*), *Enterococcus, Bacillus*, and *Escherichia* are also applied (1, 3, 4).

Due to historical and technological reasons as well as the nutritional value of milk, most probiotic foods are based on dairy products; thus, they may cause inconveniences for some groups of consumers who do not tolerate lactose and are allergic to proteins or are vegetarian. Pineapple (*Ananas comosus*) is a tropical and economically significant plant from the family *Bromeliaceae* (5). About 30 million tons of pineapple were produced worldwide in 2017 (6), and it became the third most important fruit crop in the tropical and subtropical regions of the world, only preceded by banana and citrus (7). Pineapple juice is rich in carbohydrates (13 g/100 ml), proteins (0.55 g/100 ml), vitamins—especially vitamin A (58 IU/100 ml), β-carotene (35 mcg/100 ml), vitamin C (48 mg/100 ml), vitamin K (0.73 mcg/100 ml), niacin (0.5 mg/100ml), riboflavin (0.06 mg/100 ml), thiamin (0.06 mg/100 ml), vitamin B6 (0.12 mg/100 ml), pantothenic acid (0.25 mg/100 ml), choline (5.5 mg/100 ml), and betaine (0.12 mg/100 ml)—phytosterols (0.55 mg/100 ml), in minerals such as calcium (13 mg/100 ml), iron (0.3 mg/100 ml), magnesium (12 mg/100 ml), phosphorous (8 mg/100 ml), potassium (109 mg/100 ml), sodium (1.03 mg/100 ml) zinc (0.12 mg/100 ml), copper (0.12 mg/100 ml), and manganese (0.9 mg/100 ml) (8). In addition, pineapple is also rich in phenolic compounds (9–11) such as gallic acid, chlorogenic acid, and ferulic acid, which have been shown to have antioxidative, antimutagenic, and anticarcinogenic effects and have protective roles against cardio-vascular diseases and cataracts (12). Since pineapple juice already contains beneficial nutrients, it may serve as an ideal food matrix for carrying probiotic bacteria. Furthermore, it has a very pleasing taste profile to all age groups and is perceived as being healthy and refreshing. However, these essential nutrients of pineapple juice could also limit probiotic survival in the juice (1, 13). Due to the fact that pH has a very strong effect on the survival of probiotics, especially bifidobacteria (13), the research works were generally carried out in two directions: a) fortification (without fermentation) of pineapple juice (14) or b) fermentation using a single lactic acid bacteria strain (15). There is no doubt that probiotics must survive and retain their functional features during the entire food processing operation, including storage. One important criterion is that it must contain at least 10^6^ cfu/ml of the living probiotic strain(s) at the time of consumption (16). Furthermore, the loss of probiotic viability during gastrointestinal transit, where the principal stressors are the shifting pH and bile, is also considered as a hurdle that probiotics must overcome to fulfill their biological role. Despite the fact that some studies available in the literature deal with the fermentation of pineapple juice, we still lack an understanding of the viability and survival ability of individual probiotic strains during the fermentation and storage processes. Additionally, the effects of prebiotics on the survival of probiotics and the stability of fermented pineapple juice are still not clear. This study focused on the fermentation of pineapple juice with three probiotic bacteria strains (*Bifidobacterium lactis* Bb-12, *Lactobacillus plantarum* 299V and *Lactobacillus acidophilus* La5) as well as on the survival of probiotics and the stability of the fermented beverage.

## Materials and Methods

### Pineapple Juice

The 100% pineapple fruit juice (Happy Day juice, Rauch Hungaria, Hungary) was purchased commercially from the local market. The initial pH of the culture medium was adjusted to pH 6.7 using 4N NaOH before fermentation.

### Media

The Trypticase–Phytone–Yeast medium (TPY) contained (per liter) 10 g trypticase (BBL), 5 g phytone (BBL), 5 g glucose, 2.5 g yeast extract (Difco), 1 ml Tween 80, 0.5 g L-cysteine HCl, 2 g K_2_HPO_4_, 0.5 g MgCl${}_{2}^{*}$6H_2_O, 0.25 g ZnSO${}_{4}^{*}$7H_2_O 0.25 g, 0.15 g CaCl_2_, and 0.03 g FeCl_3_. TPY agar is the TPY medium supplemented by agar–agar in a concentration of 15 g/l. The pH value of the medium was around pH 6.0.

Beeren's agar medium contained (per liter) 44 g Columbia agar (Oxoid CM331), 5 g glucose, 0.5 g L-cysteine HCl, 5 g agar–agar, and propionic acid 5 mL. The propionic acid was added to the medium after sterilization, and the final pH was adjusted to pH 5.0 with 1N NaOH. MRS agar was prepared based on a recipe given by De Man et al. (17).

#### Micro-Organisms and Their Maintenance

Probiotic strains (*Bifidobacterium lactis* Bb12, *Lactobacillus plantarum* 299V, and *Lactobacillus acidophilus* La5) were obtained from Chr. Hansen A/S (Hřrsholm, Denmark). The TPY medium and the MRS medium were used for pre-culturing bifidobacteria and lactic acid bacteria, respectively, at 37°C for 24 h. In the case of bifidobacteria, the anaerobic condition was provided by Bugbox anaerobic chamber (Ruskin Technology, USA).

### Determination of Colony Forming Units

The plate counting method (18) was applied to determine the colony-forming units during both the fermentation and storage processes. The Beeren's agar and the TPY agar as well as anaerobic conditions (in Anaerobe Jar + GasPak System or in a Bugbox anaerobic chamber) were used for incubation of bifidobacteria, while the MRS agar (Scharlau, Spain) was applied for lactobacilli. Generally, the colonies of bifidobacteria and lactic acid bacteria were counted after 72–96 h and 48–72 h, respectively.

### Fermentation Process

Fruit juice (50/100 ml flask) was inoculated with *Bifidobacterium* or *Lactobacillus* strains and kept under anaerobic conditions at 37°C using Anaerobe Jar + GasPak System (OXOID) or a Bugbox anaerobic chamber. Samples were taken at regular time intervals and the colony-forming unit (cfu) of bifidobacteria and lactobacilli were counted. In addition, the pH was measured. Also, the carbohydrate as well as lactic and acetic acid contents and antioxidant capacity of the samples were determined. A fructo-oligosaccharide, Raftiline, was added as a prebiotic to the pineapple juice at a concentration of 1% at 0, 16, and 24 h of fermentation.

### Storage Stability

After fermentation (24 h), all juices were placed in the refrigerator (4°C) for storage for 2 months. In the first month, samples were taken weekly, and in the second month twice weekly. Different analyses (viability, antioxidant capacity, survival in the stimulated gastro-intestinal (GI) conditions, fermentation capacity, etc.) were performed.

### Analysis of Carbohydrates and Organic Acids

The carbohydrate and organic acid contents of fruit juice were analyzed by the HPLC method described in a previous study (18) with one minor modification. In this study, the BioRad Aminex87H analytical column (7.7 mm × 300 mm, BioRad, USA) was applied.

### Analysis of Antioxidant Capacity

The total antioxidant capacity of the fermented pineapple juice was obtained through the ferric reducing antioxidant power (FRAP) assay described by Benzie and Strain (19). The FRAP assay measures the change in absorbance at 593 nm due to the formation of a blue-colored ferrous-tripyridyltriazine complex from a colorless oxidized ferric form by the action of electron-donating antioxidants.

Total phenolic content (TPC) was determined according to methods published by Bao et al. (20) and Jia et al. (21). The total phenolic content was expressed as mg of gallic acid equivalents (GAE) per ml of the extract.

#### Survival in GI Conditions

The probiotics were exposed for 135 min to gastric fluid (0.5% NaCl pH adjusted by 10% HCl to pH 2.0) containing pepsin at a concentration of 0.3%, followed by 2.5 h incubation in 0.05 M KH_2_PO_4_ solution (pH 7.43) in the presence of 0.6% bile salts.

### Statistical Analysis

Generally, experimental data were obtained from triplicate runs of all fermentations and duplicate analysis of samples, then were processed statistically using one-way analysis of variance (ANOVA) as well as unpaired and paired Student's *t*-tests. The Statistica v9.0 software package (StatSoft, USA) and R studio package (r-project.org) were used for data processing and only *p* < 0.05 was accepted as the statistical significance level. The mean and standard deviation (SD) of data are presented in this study.

## Results and Discussion

### Fermentation of Pineapple Juice

Generally, the pH of commercially available pineapple juices ranges from pH 3.5 to pH 4.0; thus before fermentation the pH was neutralized (pH ≈6.7) by 4 N NaOH solution. The juices were inoculated with different probiotic bacteria (*Lactobacillus acidophilus, Lactobacillus plantarum*, and *Bifidobacterium lactis*) at an initial cell density of about 10^6^-10^7^ cfu/ml. Cell counts, productivity, pH, and the contents of some sugars after 24 h of fermentation are summarized in Tables 1, 2. The juices were rich in fermentable sugars such as glucose (4.4%, w/v), fructose (2.4%, w/v), and disaccharides (7.4%, w/v). Additionally, small amounts of acetic acid and citric acid were detected. Both genera *Lactobacillus* and *Bifidobacterium* were reported to have high requirements of free amino acids, peptides, vitamins, and fermentable carbohydrates for growth (22) due to a lack of proteolytic activity (16). All investigated *Lactobacillus* and *Bifidobacterium* strains were able to grow well in the pineapple juice without supplement of any nutrients, meaning this matrix in itself was a suitable medium for propagation of probiotic bacteria. Costa et al. (15) also reported that the *L. casei* NRRL B442 strain grew very well in native pineapple juice. In the case of lactic acid bacteria (LAB), the cell numbers passed, while in the case of bifidobacteria it reached a level of 10^9^ cfu/ml after 24 of fermentation (Table 1). Interestingly, in our previous study, these probiotic bacteria exhibited better propagation properties (10^10^ cfu/ml after 24 h) in apricot juice, which contains about 6 g/100 ml fructose and 3.4 g/100 ml glucose (18). Our results from the bifidobacteria were in agreement with data reported by Havas et al. (23) when they checked the performance of some new isolates of *Bifidobacterium* in soymilk medium. Pereira et al. (24) produced probiotic cashew apple juice by fermentation with *Lactobacillus casei*, and they reported a cell density in fermented juice of about 3 × 10^8^ cfu/ml. However, the *Lactobacillus casei* NRRL B-442 grew very well in cantaloupe juice and reached about 10^10^ cfu/ml (25). In a comparison of two lactic acid bacteria strains, the cell count of the *Lactobacillus plantarum* 299V strain was significantly higher than that of the *L. acidophilus* La5 strain. This can be explained by their origins. While *L. plantarum* was isolated from a plant source, *L. acidophilus* originated from animal source, thus *L. plantarum* should grow better in a plant matrix than *L. acidophilus*.

**Table 1:** Cell yield, productivity and change of pH during pineapple juice fermentation supplemented by prebiotic.

	**Time of prebiotic supplementation**	**Strains**		
		***L. acidophilus* La5**	***L. plantarum 299V***	***B. lactis* Bb12**
pH	No prebiotic	3.8	3.9	5.6
	8 h	3.8	3.9	4.4
	24 h	3.8	3.8	4.7
Cell count (cfu/ml)	No prebiotic	3.35*10^9^	8.41*10^9^	9.68*10^8^
	8 h	5.46*10^9^	7.09*10^9^	1.46*10^9^
	24 h	3.99*10^9^	4.85*10^9^	5.15*10^8^
Productivity (cfu/ml*h)	No prebiotic	1.38*10^8^	3.49*10^8^	4.02*10^7^
	8 h	2.25*10^8^	2.94*10^8^	6.08*10^7^
	24 h	1.64*10^8^	2.01*10^8^	2.14*10^7^

**Table 2:** Change of carbohydrate content during pineapple juice fermentation supplemented by prebiotic.

	**0 h**	***L. acidophilus*** **La5**			***L. plantarum 299V***			***B. lactis*** **Bb12**		
		**No prebiotic**	**8 h**	**24 h**	**No prebiotic**	**8 h**	**24 h**	**No prebiotic**	**8 h**	**24 h**
	**Carbohydrate content (g/100 ml)**									
Trisaccharide	0.40	0.34	0.38	–	0.40	0.34	–	0.20	0.23	0.01
Disaccharide	2.36	2.13	2.22	2.25	2.50	2.18	2.76	1.53	1.87	1.79
Glucose	2.94	2.16	2.26	2.33	2.55	2.30	2.84	2.14	2.26	2.57
Fructose	2.65	1.75	1.82	1.78	2.40	2.18	2.43	0.33	0.45	–

Volumetric productivities of cell yields of *L. acidophilus* La5, *L. plantarum* 299V, and *B. lactis* Bb-12 were 1.38 × 10^8^, 3.49^*^10^8^, and 4.02^*^10^7^ cfu/ml^*^h, respectively. These results were lower than data reported by Bujna et al. (18) when they produced beverage of apricot juice by mono and mixed cultural fermentation with bifidobacteria and lactic acid bacteria (LAB). However, our results agree with data reported by Kun et al. (26) as well as Fonteles et al. (25).

During the fermentation process, some short chain fatty acids (SCFA) were produced in decreasing pH values. In the case of bifidobacteria, the pH dropped from pH 6.7 to pH 5.0 at 16 h and to pH 4.8 at 24 h, while in the case of LAB, the pH dropped to about pH 3.8 at 24 h of fermentation. Lactic acid bacteria produced about 150 mM lactic acid and 30 mM acetic acid in fermented pineapple juice. These values were higher than those reported by Bujna et al. (18) when they did the fermentation of apricot juice by similar LAB strains. Our results are also higher than those published by Gardner et al. (27) as well as by Di Cagno (28). The ratios of lactic acids to acetic acids in *L. plantarum* 299V and *L. acidophilus* La5 were 5.37 and 9.91, respectively, and were not affected significantly by supplementation with prebiotic carbohydrate (Table 3). Production of organic acids by *Lactobacillus* may depend on the composition of the medium applied (29). Zalán et al. (30) confirmed that some strains of *Lactobacillus* can change their fermentative profile from homofermentative to mix-acid fermentation depending on the composition of media. In our cases, both nitrogen and carbon sources in pineapple juice were sufficient for LAB, and thus very good molar ratios of lactic acid to acetic acid were obtained. The presence of lactic acid generally results in good taste for fermented beverages.

**Table 3:** Change of organic acid content during pineapple juice fermentation supplemented by prebiotic.

**Strains**	**Organic acids**	**Concentration of organic acids (μmol/ml)**		
		**Time of prebiotic supplementation**		
		**No prebiotic**	**8 h**	**24 h**
*L. acidophilus* La5	Lactic acid	170.08	154.71	138.39
	Acetic acid	31.66	25.31	28.07
	Molar ratio of lactic acid to acetic acid	5.37	6.11	4.93
*L. plantarum* 299V	Lactic acid	166.76	144.17	149.15
	Acetic acid	16.83	14.49	14.84
	Molar ratio of lactic acid to acetic acid	9.91	9.95	10.05
*B. lactis* Bb12	Lactic acid	6.97	14.77	6.19
	Acetic acid	23.06	20.86	22.66
	Molar ratio of lactic acid to acetic acid	0.30	0.71	0.27

In the case of *Bifidobacterium lactis* Bb-12, a higher concentration of acetic acid was detected. The concentrations of lactic acid and acetic acid were 6 and 23 mM in natural juices, 15 and 21 mM in the case of supplementation with prebiotic at 16 h, and 7 and 23 mM in the case of supplementation with prebiotic at the initiation of fermentation, respectively. The highest amount of lactic acid (15 mM) was produced when the prebiotic was added at 16 h fermentation. It is well-known that *Bifidobacteria* ferment glucose via the so-called “bifidus” pathway, in that the phosphoketolase plays a key enzyme role. Theoretically, from 1 molecule of glucose, 1 molecule of lactic acid and 1.5 molecule acetic acid will be liberated (31). Our results (with the exception of one case) did not fit this theory, but we think it depends on the actual fermentable sugar spectrum and on sugar concentration as well as on the amount of nitrogen in the fermentation medium. Unfortunately, this mechanism is still unclear, and more studies should be carried out. It is well-known that a high concentration of acetic acid (metabolic activity of bifidobacteria) may cause the odor and vinegar-like taste of products that is unacceptable.

In the case of the *B. lactis* Bb-12 strain, the concentration of glucose, fructose, and disaccharides decreased after 24 h of fermentation, inferring intensive hydrolysis of disaccharides and release of monosaccharides. The concentrations of glucose and fructose at 24 h when prebiotic was added at the start of the fermentation were 2.14 g/100 ml and 0.33 g/100 ml, respectively. The concentration of fructose was zero in the case of the prebiotic added at the end of the fermentation. Some papers reported that many bifidobacteria strains are able to utilize disaccharides especially sucrose natively (18, 26). In the case of *L. acidophilus* La5, the concentration of fructose decreased drastically after 24 h of fermentation, while the concentration of glucose and disaccharides slightly decreased. Generally, *L. acidophilus* prefers glucose to fructose, and it is a homofermentative organism that utilizes glucose through the Embden Meyerhof Parnas (EMP) pathway (32). Our results showed that in a complex medium like pineapple juice, with the presence of several fermentable sugars and other compounds, the order of utilization of sugars may be changed. In this case, the preference order should be fructose > glucose ≥ sucrose. Bujna et al. (18) reported an order of disaccharide > glucose > fructose with apricot juice fermented by *L. acidophilus* La5 and *L. casei* 01 strains. In the case of *Bifidobacterium lactis* Bb12, the utilization rate of fructose was higher than those of glucose and disaccharides. However, this result is in disagreement with data reported by Kun et al. (26) and, in general, bifidobacteria prefers glucose to fructose and other sugars.

The concentrations of TPC and FRAP in pineapple were determined to be 0.4 mg/ml gallic acid and 2.66 mM FeSO_4_/ml equivalents, respectively. Our results are in agreement with data reported by Lu et al. (33) when they studied the physico-chemical properties, antioxidant activity, and mineral contents of 26 pineapple genotypes grown in China. The fermentation of pineapple juice with *L. acidophilus* La 5, *L. plantarum* 299V, and *Bifidobacterium lactis* Bb12 strains caused slight changes (increase) in the concentrations of TPC and FRAP. These values (at 24 h fermentation) were about 0.45 mg/ml gallic acid and 3.0 mM FeSO_4_/ml equivalents, respectively. Bujna et al. (18) recorded a slight increase in antioxidant capacity during fermentation of apricot juice with *B. lactis* Bb12 and *L. acidophilus* La5. Martin and Matar (34) also registered an increase in antioxidant activity of blueberry juice during fermentation with a novel bacterium from the fruit microflora *Serratia raccinii*. Probiotics can produce various metabolites with antioxidant activity such as glutathione, butyrate, folate, etc. (35, 36), but the generally accepted opinion is that the antioxidative properties of probiotic bacteria are specific features of individual strains (18).

After fermentation, the samples were centrifuged, and the collected cell mass was submitted to investigate the effects of stress factors on the survival of probiotic bacteria. All investigated probiotic bacteria exhibited very good viability against both stresses: 0.3% pepsin and 0.6% bile salts. Only a 0.01 log cfu/ml loss was detected after treatment with 0.3% pepsin for 135 min, while these probiotic bacteria kept all viable cells after further treatment with 0.6% bile salts for 2.5 h. Some authors also reported the resistance of fresh probiotic bacteria against gastric stresses (37, 38).

### Storage Stability of Fermented Pineapple Juice

**Figure 1:**
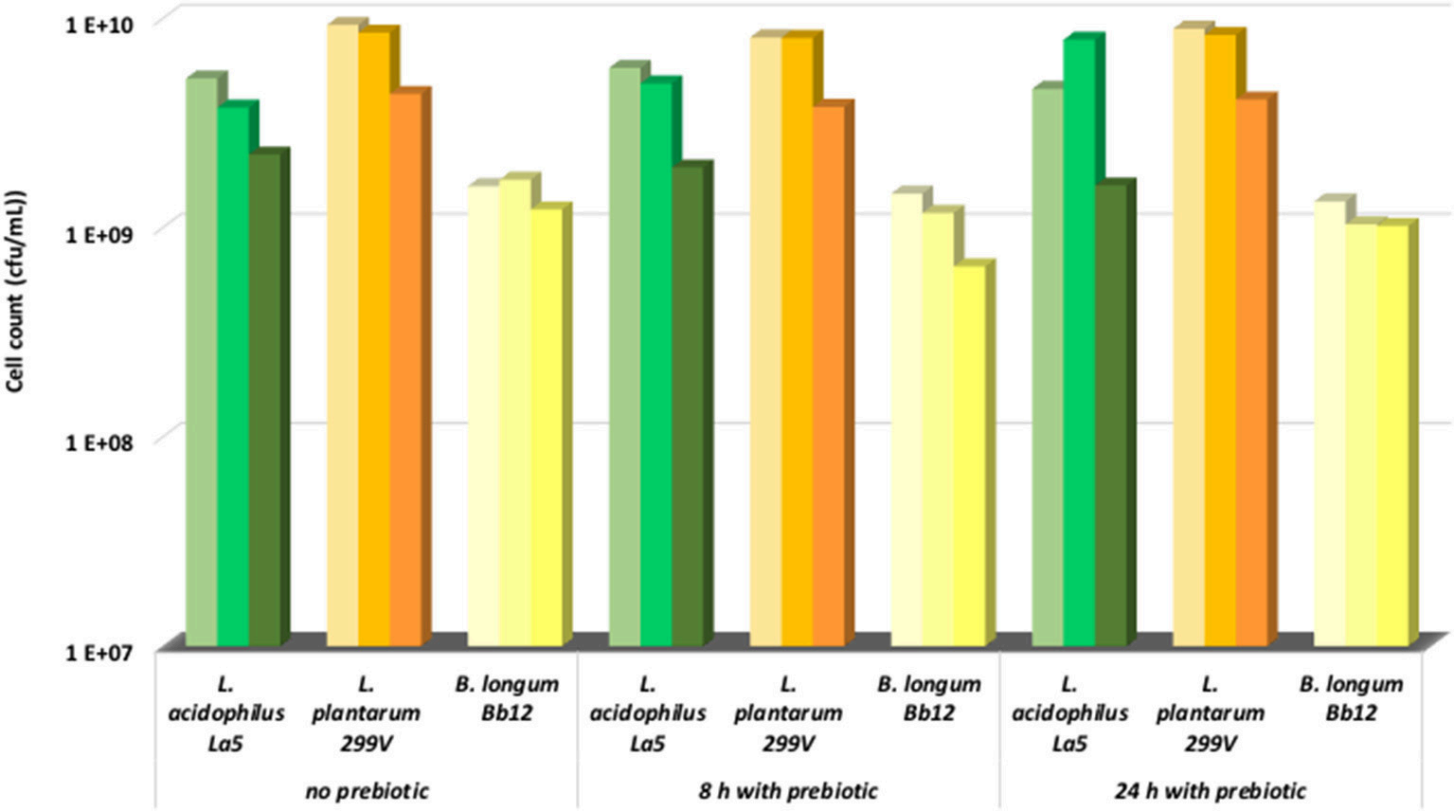
Change of cell count during storage at 4°C (1^st^ column, 0 month; 2^nd^ column, 1 month; 3^rd^ column, 2 months).

The storage of the pineapple beverage was carried out with and without prebiotics. In all cases, the microbial population did not change significantly in the first month. The cell counts of lactobacilli were approximately in the range of 10^9^ and 10^10^ cfu/ml, while the cell numbers of bifidobacteria were in the range of 10^8^ and 10^9^ cfu/ml (Figure 1). During this period, minimal changes in the pH values of beverages were detected (data not shown). This is explained by the high buffering capacity of fermented pineapple juice. Additionally, our results also show that supplementation of prebiotic fructo-oligosaccharides did not have any effects on the viability of probiotic bacteria. Oligofructose was also reported to have no influence on the physicochemical characteristics (pH, titratable acidity, color, and turbidity), acceptability, purchase intent, or storage stability of the clarified apple juice products (39, 40), thus our results completely fit with this opinion. Champagne and Gardner (37) evaluated the viability of nine strains of the *Lactobacillus* genus in a commercial fruit drink stored at 4°C for up to 80 days. The pH of the drink was pH 4.2, which enabled good stability of many cultures during storage. They found that the viability of the *L. plantarum* was excellent, meaning almost viable cells were retained after 80 days storage, whereas the viability of *L. acidophilus* was the worst. More than 80% of viable cells were lost after 80 d storage in this medium. *L. plantarum* is originated from plant source, thus this species held a better level of viability in the fruit juice. Daneshi et al. (14) found that *L. acidophilus* La5, *L. plantarum* and *B. lactis* Bb-12 strains lost a minimal number of cell counts (<10%) during storage at 4°C for 20 days in non-fermented milk and a carrot juice mix drink. Other studies (41–43), however, reported the decrease (about 3–4 log cfu/ml) in viability of probiotic strains incorporated into different juices such as orange juice, grape juice, passion fruit juice, pineapple juice, cranberry juice, and natural cornelian cherry juice during storage. There is no doubt that the viability of LAB varied from strain to strain, but it also strongly depends on the nature and quality of the carrier-matrix. Organic acids and flavor compounds—especially in non-fermented matrices—have negative effects on the survival of probiotic LAB (14). In our case, the fermentation definitely contributed to the viability of probiotic bacteria.

In the first month of storage at 4°C in refrigerated conditions, the total phenolic content and antioxidant capacity decreased to 0.4 mg/ml gallic acid and 2.7 mM/ml FeSO_4_ equivalents, and they remained stable for next month. Patthamakanokporn et al. (44) also reported the decrease in antioxidant activity and total phenolic compounds during storage of selected fruits. The decrease in antioxidant activity as well as phenolic and anthocyanin contents may be due to the slight activity of probiotic bacteria in refrigerated temperatures as well as the presence of dissolved oxygen in samples, which resulted in oxidation of phenolic compounds (41). It has been reported that in the absence of light and oxygen, the number of phenolic compounds did not change considerably during the refrigerated storage period (44).

The survival of probiotic cells in fermented pineapple juice against stress factors was modeled and investigated. After storage of fermented pineapple juice for 1 month, the probiotic bacteria lost about 0.11 log cfu/ml viability after treatment with 0.3% pepsin for 135 min, and a further 0.1 log cfu/ml after treatment with 0.6% bile salts. These values were higher by a factor of 10 than data from the fresh fermented pineapple juice. Champagne and Gardner (37) found that the viability of fresh probiotic culture was not affected by the presence of 0.3% bile salts or of pancreatic enzymes. However, the cultures that were stored for 35 days at 4°C in the fruit drink had on average 1.2 log more viability losses than the fresh cultures when exposed to 2 h incubation at pH 2.0 to simulate gastric stress. Stored probiotic cultures in fruit juices seem to be less resistant to acidic conditions than fresh cells; thus, this should be a new research topic in the development of probiotic pineapple juice drinks.

## Conclusion

Pineapple in itself is a good substrate for the growth of probiotic bacteria *Lactobacillus* and *Bifidobacterium* strains, thus it should be a potential alternative functional food matrix. The characteristics of fermented beverages are strongly dependent on the spectrum of actual fermentable sugars, on the probiotic strain(s), and on nitrogen (protein) content. Supplementation with prebiotic fructooligosaccharides during the fermentation process increased the production of lactic acid by bifidobacteria and slightly improved the stability of fermented and probiotic cells. The role of nitrogen sources (both quality and quantity) should be checked to improve the sensorial taste of pineapple juice beverage. Overall, probiotic *Lactobacillus plantarum* 299V is suggested to be used for the development of probiotic pineapple juice drinks.

## Author Contributions

EB, JR-S, QN, and RP developed the idea, evaluated the data, and wrote the manuscript. BN, NF, and AT carried out experiments and collected data. All authors proofread and approved the final manuscript.

### Conflict of Interest Statement

The authors declare that the research was conducted in the absence of any commercial or financial relationships that could be construed as a potential conflict of interest.
